# Basal release of 6-cyanodopamine from rabbit isolated aorta and its modulation of vascular reactivity

**DOI:** 10.1007/s00210-026-05474-8

**Published:** 2026-05-30

**Authors:** Eric Xavier Dos Santos, Kashif Ali, José Britto-Júnior, Larryn Peterson, Joshua J. L. Morris, Alex Miller, Felipe Fernandes Jacintho, Maria Elisabete Moraes, Vincenzo Santagada, Giuseppe Caliendo, Beatrice Severino, Edson Antunes, Gilberto De Nucci

**Affiliations:** 1 Department of Pharmacology, Faculty of Medical Sciences, State University of Campinas (UNICAMP), 126 Tessália Vieira de Camargo St, Campinas, Sao Paulo, 13083-887 Brazil; 2 Department of Chemistry, Rhodes College, Memphis, TN USA; 3 Clinical Pharmacology Unit, Drug Research and Development Center, Federal University of Ceará, Fortaleza, Ceará Brazil; 4 Department of Pharmacy, School of Medicine, University of Naples Federico II, Naples, Italy; 5 Department of Pharmacology, Institute of Biomedical Sciences, University of São Paulo (USP), São Paulo, Brazil; 6 Department of Pharmacology, Faculty of Medicine, São Leopoldo Mandic, Campinas, São Paulo, Brazil

**Keywords:** Cyanide, 6-Nitrodopamine, Acetylcholine, Soluble guanylyl cyclase, Nitric oxide

## Abstract

6-Cyanodopamine (6-CYD) is a recently identified endogenous catecholamine that exerts positive chronotropic and inotropic actions, but its occurrence in vascular tissue and effects on vascular reactivity are unknown. In endothelium-intact and endothelium-denuded rabbit isolated aortic rings, we investigated the basal release of 6-CYD and its effects on aortic smooth muscle reactivity. 6-Cyanodopamine release was quantified by LC–MS/MS. Isometric tension was recorded to assess concentration–response curves to noradrenaline, adrenaline, and dopamine in aortic rings pre-incubated or not with 6-CYD, whereas vasorelaxation was evaluated in endothelin-1 (ET-1)-pre-contracted rings. Rabbit aorta released significant amounts of 6-CYD, which were largely reduced either by endothelium removal or pre-incubation with the NOX1/4 inhibitor GKT137831 (1 µM), whereas the nitric oxide (NO) synthase inhibitor L-NAME (100 µM) and the voltage-gated sodium channel blocker tetrodotoxin (TTX, 1 µM) had no significant effect. Addition of 6-CYD (0.1 pM to 10 nM) had no contractile activity itself, but it significantly potentiated noradrenaline- and adrenaline-induced aortic contractions, an effect largely reduced by endothelium removal. The dopamine-induced aortic contractions remained unaffected by 6-CYD. In ET-1-pre-contracted rings, 6-CYD (1 nM–10 µM) produced concentration-dependent relaxations that were significantly reduced by endothelium removal and L-NAME (100 µM), as well as by the soluble guanylyl cyclase (sGC) inhibitors ODQ (100 µM) and methylene blue (10 µM). In conclusion, these findings identify vascular endothelium as a source of 6-CYD and indicate this catecholamine as a novel endogenous modulator of vascular tone, acting through a dual mechanism, namely, potentiating vasoconstriction and producing NO/sGC-dependent vasorelaxation.

**Supplementary Information:**

The online version contains supplementary material available at 10.1007/s00210-026-05474-8.

## Introduction

Cyanide is a naturally occurring substance in the environment and is traditionally recognized as an environmental toxin (Petrikovics et al. 2015), commonly associated with poisoning and chemical weapons (Yang et al. 2022). In healthy volunteers, cyanide levels vary from 0.3 to 8 µM (Uwakwe et al. 1991; Fasco et al. 2011) and are apparently increased in smokers (Vinnakota et al. 2012). Hydrogen cyanide can be produced by mammalian cells from the amino acid glycine in a reaction modulated by peroxidases (Borowitz et al. 1997; Gunasekar et al. 2000; Stelmaszynska, 1986; Zuhra et al. 2025). Chlorination of glycine by myeloperoxidase-H_2_O_2_-Cl^−^ system generates N-monochloroglycine and hydrogen cyanide (HCN; Zgiczynski and Stelmaszynska, 1979). Cyanide causes vascular responses through mitochondrial inhibition, modulation of calcium signalling, and interference with the soluble guanylyl cyclase/cyclic guanosine monophosphate pathway (Alan-Albayrak and Simonsen 2025).

6-Cyanodopamine (6-CYD), the first identified endogenous mediator containing cyanide, is a novel catecholamine initially identified in rabbit isolated atria and ventricles (Júnior et al. 2023), and then in rat isolated heart (Britto-Júnior et al. 2025), rat (Dal Pozzo et al. 2024) and human (Britto-Júnior et al. 2026a) isolated vas deferens, and also in the plasma of patients with chronic kidney disease (Ribeiro et al. 2024). The basal release of 6-CYD in the rat isolated heart is sensitive neither to pre-treatment with the nitric oxide (NO) synthase inhibitor L-NAME (Rees et al 1990) nor to the voltage-gated sodium channel inhibitor tetrodotoxin (TTX; Narahashi et al. 1964), despite its levels being significantly decreased by either the NOX1,4 inhibitor GKT137831 (Jiang et al. 2012) or hydrogen peroxide (H_2_O_2_). In addition, in the rat isolated heart, 6-CYD itself has potent positive chronotropic and inotropic effects, being also able to significantly potentiate the positive chronotropic and inotropic effects induced by noradrenaline (Britto-Júnior et al. 2025). Interestingly, in rat and human vas deferens smooth muscle, 6-CYD was devoid of contractile activity; however, it significantly potentiated the contractions induced by the classical catecholamines dopamine, noradrenaline, and adrenaline, and by electric-field stimulation (Dal Pozzo et al. 2024; Britto-Júnior et al. 2026a). Although the actions reported above are similar to those described for 6-nitrodopamine (6-ND) (Zatz and De Nucci 2024), in the isolated vas deferens, 6-ND and 6-CYD presented a co-synergism, suggesting that they are not acting on the same receptor or via the same transduction mechanism.

Endothelium-derived 6-ND is the most potent endogenous vasorelaxant agent of the rabbit isolated aorta and the main mechanism by which NO relaxes this tissue (Santos et al. 2024). Since 6-CYD shares the same characteristics of 6-ND in the heart and non-vascular smooth muscle, we investigated whether the rabbit isolated aorta releases 6-CYD and whether this novel catecholamine modulates vascular smooth muscle reactivity.

## Material and methods

### Animals

Male New Zealand White rabbits (*Oryctolagus cuniculus*; *n* = 96; 2.5–3.0 kg) were obtained from Anilab (Paulínia, São Paulo, Brazil). All experimental procedures were approved by the Institutional Animal Care and Use Committee (CEUA/UNICAMP, protocol no. 6287–1/2023) and were conducted in accordance with international guidelines for the care and use of laboratory animals.

### Basal release of 6-cyanodopamine (6-CYD)

Animals were anesthetized with ketamine/xylazine (80/10 mg/kg, intramuscularly; respectively) following induction with propofol (30 mg/kg, intravenous). Euthanasia was performed by exsanguination. The aorta was removed and dissected, and the thoracic aorta was suspended separately in an organ bath (3 mL) containing Krebs–Henseleit’s solution (KHS), continuously bubbled with 95% O_2_/5% CO_2_ at 37 °C for 30 min. An aliquot (2 mL) of KHS was collected and stored at − 20 °C until analysis by liquid chromatography coupled to tandem mass spectrometry (LC–MS/MS; Campos et al. 2021), as described below. When required, 6-CYD levels were quantified in endothelium-denuded preparations. The endothelium-intact isolated thoracic aorta rings were incubated (30 min) in the absence and presence of the NO synthesis inhibitor N^ω^-nitro-L-arginine methyl ester (L-NAME, 100 µM) or the voltage-gated sodium channel inhibitor tetrodotoxin (TTX, 1 μM).

### Determination of 6-CYD levels by liquid chromatography coupled to tandem mass spectrometry (LC–MS/MS)

6-Cyanodopamine was extracted from the KHS using Strata™-X 33-mm Polymeric Reversed SPE cartridges. The LC–MS/MS analysis was performed in a single chromatographic run; the mobile phase perfused a LC ADVp Liquid Chromatography Shimadzu System (Shimadzu Corp., Kyoto, Japan) coupled to a Shimadzu 8060 triple quadrupole mass spectrometer operating in ESP + mode at 350 µL/min. The dissolved residues were injected by a SIL-30AC autoinjector, at 8 °C. In contrast to the original method (Júnior et al. 2023), two transitions were employed to enhance selectivity: one transition employed as the quantifier transition and the other employed as the qualifier transition (Ribeiro et al. 2024).

### Preparations for isometric tension recordings

The thoracic aorta rings (5-mm length) were suspended vertically between two metal hooks in 10-mL organ baths containing KHS, gassed with a mixture of 95% O_2_ and 5% CO_2_ (pH 7.4) at 37 °C. Isometric force was recorded using a PowerLab 400TM data acquisition system (Software Chart, version 7.0, AD Instrument, USA). The tissues were allowed to equilibrate for 1 h before starting the protocols, as detailed below.

### Evaluation of endothelium integrity

Endothelium-intact thoracic aortic rings were pre-contracted with phenylephrine (1 μM). After the contraction had reached a plateau, the endothelium integrity was evaluated through acetylcholine-induced relaxation (1 μM). Relaxation larger than 70% was considered indicative of endothelium integrity. When required, removal of the thoracic aorta endothelium was performed by gently rubbing the vessels with forceps. Relaxation to acetylcholine in the denuded preparations was nearly abolished, confirming the efficacy of endothelium removal.

### Effect of 6-CYD on noradrenaline-, adrenaline-, and dopamine-induced contractions in endothelium-intact and endothelium-denuded aortic rings

Aortic rings with intact endothelium (E +) were mounted for isometric tension recording and allowed to equilibrate before experimentation. Rings were pre-incubated for 30 min with vehicle (H_2_O, 100 μL) or 6-CYD. For the noradrenaline protocols, 6-CYD was tested at concentrations of 0.1, 1, and 10 pM, whereas those for adrenaline, concentrations of 1, 10, and 100 pM were used. For the dopamine protocols, 6-CYD concentrations of 0.1, 1, and 1 nM were used. Following the pre-incubation period, cumulative concentration–response curves to the respective agonist (10 nM–300 µM) were constructed with continuous recording of isometric tension.

In separate experiments, aortic rings without endothelium (E-) were pre-incubated for 30 min with vehicle or 6-CYD. For noradrenaline protocols, 6-CYD was tested at concentrations of 10 and 100 pM, whereas those for adrenaline, concentrations of 100 pM and 1 nM were used. For the dopamine protocols, 6-CYD concentrations of 1 and 10 nM were used. Cumulative concentration–response curves (10 nM–300 µM) were then constructed under isometric tension recording.

Contractile responses in all experiments were analyzed by comparing maximal effect (*E*_max_) and agonist sensitivity (pEC₅₀) with the corresponding vehicle (H_2_O, 100 μL)-treated controls.

### Relaxation responses to 6-CYD in aortic rings

Concentration–response curves to 6-CYD (1 nM–10 µM) were obtained in aortic rings pre-contracted with endothelin-1 (ET-1, 10 nM). Responses were compared between rings with endothelium-intact (E⁺) and endothelium-denuded rings (E⁻). To assess the role of the NO/cGMP pathway, tissues were pre-incubated for 30 min with one of the following inhibitors before obtaining 6-CYD curves: L-NAME, a nitric oxide synthase inhibitor (100 µM); ODQ, a heme-site inhibitor of soluble guanylyl cyclase (100 µM); or methylene blue, a non-specific soluble guanylyl cyclase inhibitor (10 µM). In a separate set of experiments, the aortic rings were pre-contracted with endothelin-1 (ET-1, 10 nM). After reaching a stable contractile plateau, 6-CYD at 1 µM was added, and changes in vascular tone were continuously recorded to evaluate relaxation. Time-control experiments were performed in rings exposed to ET-1 (10 nM) alone, without the addition of 6-CYD.

### Chemicals and reagents

6-CYD was synthesized at Rhodes College (Rote et al. 2017). L-NAME and methylene blue were obtained from Sigma-Aldrich (St. Louis, MO, USA). Adrenaline, dopamine, GKT137831, noradrenaline, TTXEndothelial cells expre, and ODQ were obtained from Cayman Chemical (Ann Arbor, MI, USA). Strata™-X polymeric reversed-phase SPE cartridges (33 µm) were purchased from Phenomenex (CA, USA), and GIST-HP C18 columns were obtained from Shimadzu (Duisburg, Germany). Calcium chloride (CaCl_2_), dextrose, magnesium sulfate (MgSO_4_), potassium chloride (KCl), sodium bicarbonate (NaHCO_3_), potassium phosphate monobasic (KH_2_PO_4_), and sodium chloride (NaCl) were purchased from Merck KGaA (Darmstadt, Germany). Acetonitrile and methanol were obtained from J.T. Baker (Phillipsburg, NJ, USA), and formic acid from Mallinckrodt (St. Louis, MO, USA). Krebs–Henseleit solution (KHS) contained (mM) 118 NaCl, 4.7 KCl, 2.5 CaCl_2_, 1.2 MgSO_4_, 25 NaHCO_3_, 1.2 KH_2_PO_4_, and 5.6 dextrose.

### Data analysis

Nonlinear regression analysis to determine pEC_50_ was performed using GraphPad Prism (GraphPad Software, version 10), with the constraint that *F* = 0. All concentration–response data were evaluated for a fit to a logistic function in the form: *E* = *E*_max_/([1 + (10^c/10^x)]^*n* + *F*), where *E* represents the increase in contractile response induced by the agonist, *E*_max_ is the maximum effect of the agonist, *c* is the logarithm of the concentration of the agonist that produces 50% of *E*_max_, *x* is the logarithm of the drug concentration, *n* is the exponential term that defines the slope of the concentration–response curve, and *F* is the response observed in the absence of the added drug. Data are presented as means ± SEM. Comparisons between two independent groups were performed using an unpaired Student’s *t*-test, and differences were considered statistically significant when *p* < 0.05.

## Results

### Basal release of 6-CYD

**Fig. 1:**
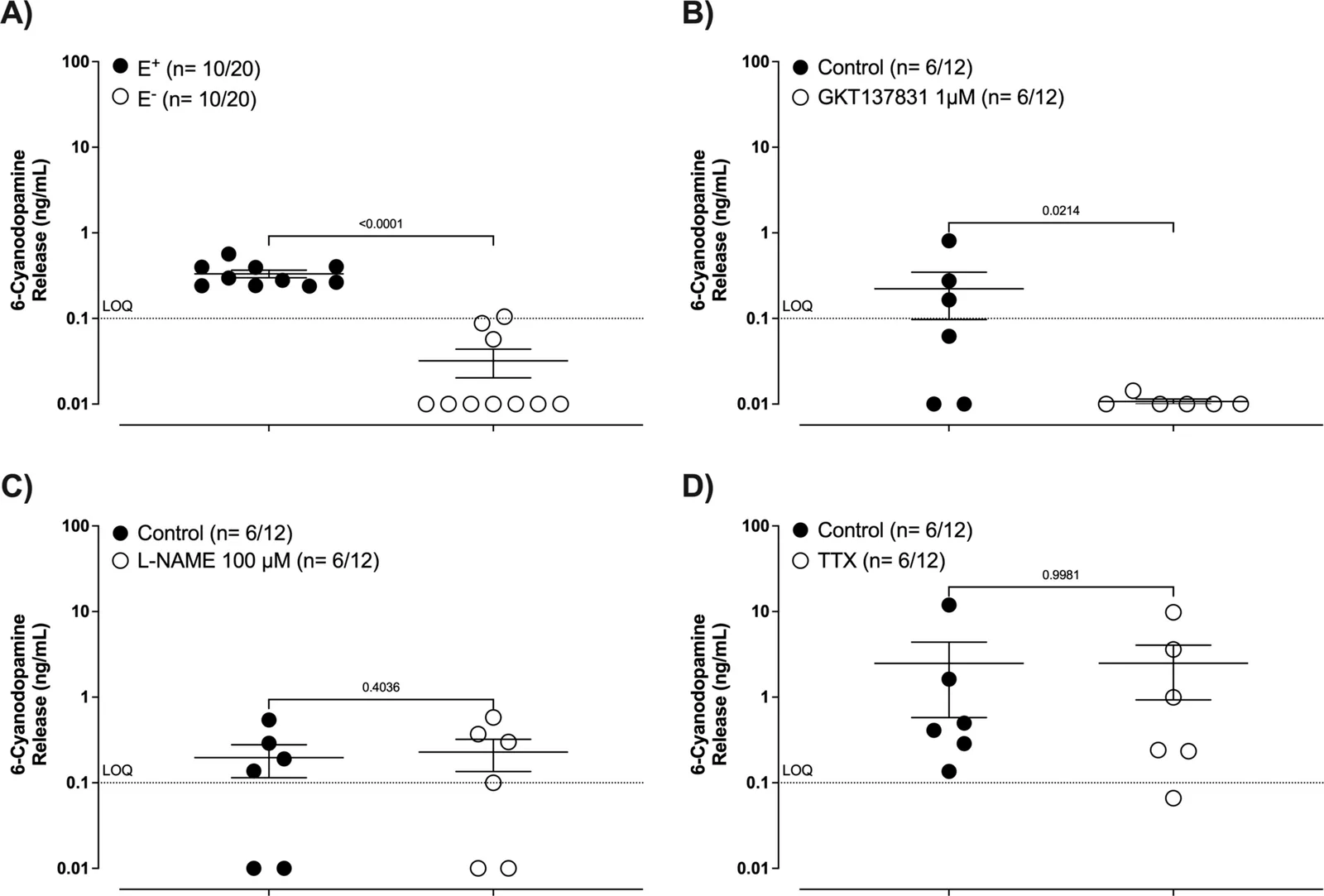
Release of 6-cyanodopamine (6-CYD) from rabbit isolated aorta. The release of 6-CYD was quantified by liquid chromatography–tandem mass spectrometry (LC–MS/MS) in endothelium-intact (E^+^) and endothelium-denuded (E^−^) preparations (**A**). The effects of pre-incubation for 30 min with GKT137831 (1 µM), N^ω^-nitro-L-arginine methyl ester (L-NAME, 100 µM), and tetrodotoxin (TTX, 1 µM) on 6-CYD release are shown (**B**–**D**, respectively). Each point represents an individual experiment, and the horizontal lines indicate mean ± SEM. The number of experiments is indicated by *x*/*y* where *x* represents the number of analyses and *y* the number of animals. LOQ, limit of quantification. Student’s *t*-test related to number of analyses or animals was used to compare groups. LOQ, limit of quantification

Quantification by LC–MS/MS demonstrated that 6-CYD is released from endothelium-intact rings of rabbit isolated aorta under control conditions. Removal of the endothelium (E^−^) significantly reduced the basal 6-CYD release compared with endothelium-intact rings (E⁺; Fig. 1A). Pre-incubation of endothelium-intact rings with the NADPH oxidase (NOX1/4) inhibitor GKT137831 (1 µM, 30 min) abolished the basal 6-CYD release (Fig. 1B). Neither L-NAME (100 µM, 30 min; Fig. 1C) nor tetrodotoxin (TTX, 1 µM, 30 min; Fig. 1D) modified the basal release of 6-CYD (Fig. 1D).

### Potentiation by 6-CYD of noradrenaline- and adrenaline-induced contractions in aortic rings with intact endothelium

**Fig. 2:**
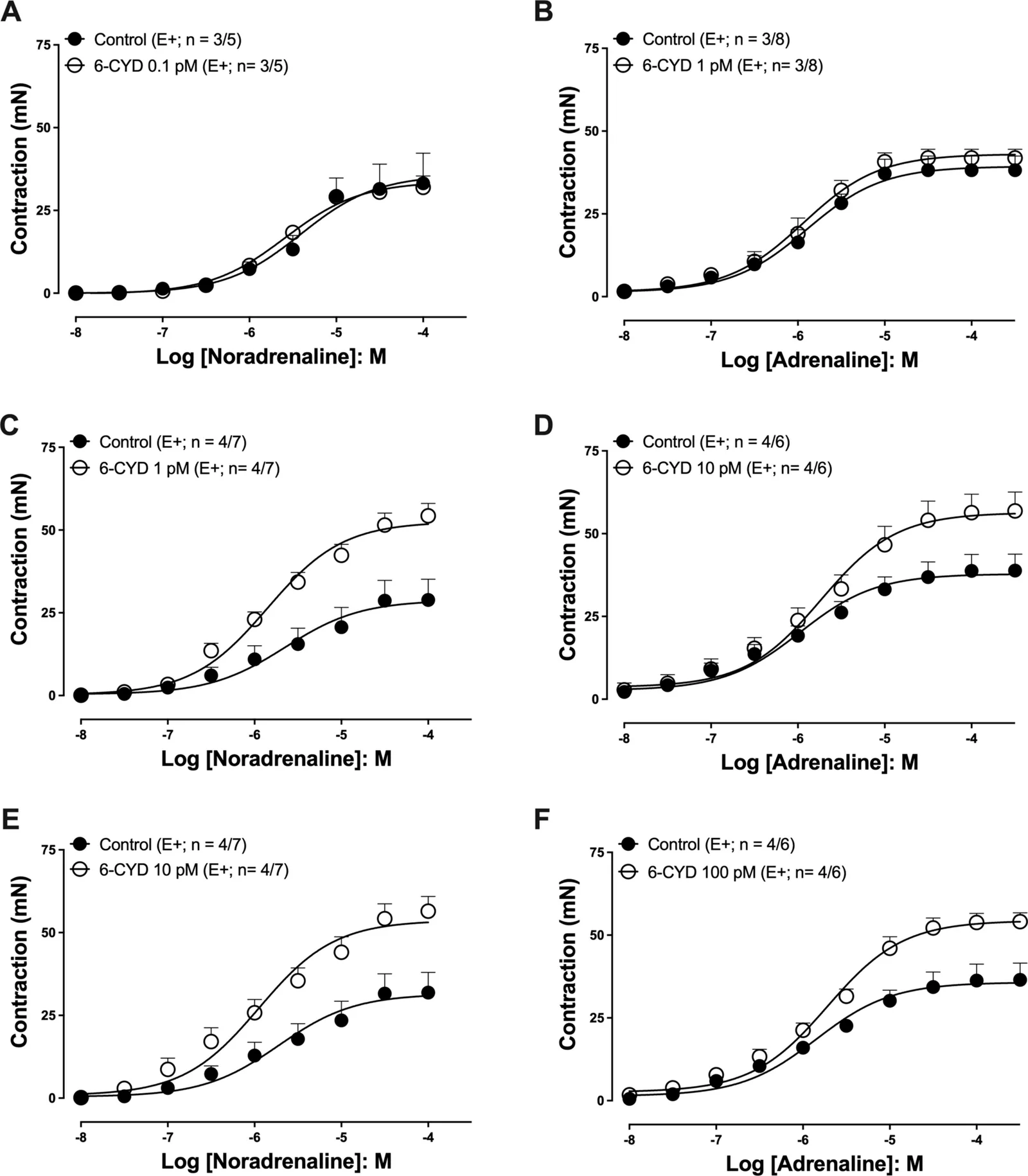
Effects of 6-cyanodopamine (6-CYD) on noradrenaline- and adrenaline-induced contractions in endothelium-intact (E^+^) rabbit aortic rings. Concentration–response curves to noradrenaline (**A**–**C**) and to adrenaline (**D**–**F**). Rings were incubated for 30 min with 6-CYD at 0.1 (**A**), 1 (**B**, **C**), 10 (**D**, **E**), or 100 pM (**F**). Control responses were obtained in the absence of 6-CYD. Data are expressed as mean ± SEM. The number of experiments is indicated by *x*/*y* where *x* represents the number of animals and *y* the number of rings

6-Cyanodopamine up to 300 μM failed to induce contraction per se in endothelium-intact aortic rings, even in the presence of L-NAME (100 μM, Figure S1). Noradrenaline (0.01–100 μM) elicited concentration-dependent contractions in all experimental groups (Fig. 2A, C, and E). Pre-incubation of the rings with 6-CYD at 0.1 pM for 30 min produced no modification of the noradrenaline concentration–response curve (Fig. 2A). In contrast, pre-incubation with 6-CYD at 1 pM (Fig. 2C) and 10 pM (Fig. 2E) significantly potentiated noradrenaline-induced contractions, as evidenced by an increase in the maximal contractile response (*E*_max_) compared with control tissues, without significantly changing the pEC_50_ values (Table 1).

**Table 1:** Potency (pEC₅₀) and maximal responses (*E*_max_) to noradrenaline, adrenaline, and dopamine in endothelium-intact (E +) and endothelium-denuded (E-) rabbit aortic rings in the absence (control) and presence of different concentrations of 6-cyanodopamine (6-CYD)

	pEC_50_ (log[M])	*E*_max_ (mN)	*N*
**Endothelium-intact (E +)**			
**Noradrenaline**			
(E +) control	5.45 ± 0.10	33.18 ± 9.15	3/5
(E +) 6-CYD (0.1 pM)	5.59 ± 0.10	31.97 ± 3.45	3/5
(E +) control	5.59 ± 0.22	28.88 ± 6.30	4/7
(E +) 6-CYD (1 pM)	5.86 ± 0.10	54.81 ± 4.40*	4/7
(E +) control	5.64 ± 0.20	31.88 ± 6.09	4/7
(E +) 6-CYD (10 pM)	5.89 ± 0.13	56.44 ± 5.10*	4/7
**Adrenaline**			
(E +) control	5.90 ± 0.12	39.33 ± 1.60	3/8
(E +) 6-CYD (1 pM)	5.93 ± 0.10	43.25 ± 1.30	3/8
(E +) control	5.99 ± 0.22	38.86 ± 4.95	4/6
(E +) 6-CYD (10 pM)	5.77 ± 0.10	56.88 ± 5.68*	4/6
(E +) control	6.04 ± 0.25	36.52 ± 5.03	4/6
(E +) 6-CYD (100 pM)	5.76 ± 0.15	55.35 ± 2.86*	4/6
**Dopamine**			
(E +) control	5.14 ± 0.13	35.10 ± 6.55	3/7
(E +) 6-CYD (100 pM)	5.17 ± 0.16	35.02 ± 5.23	3/7
(E +) control	5.15 ± 0.14	35.10 ± 6.55	3/7
(E +) 6-CYD (1 nM)	5.21 ± 0.15	31.85 ± 5.18	3/7
**Endothelium-denuded (E-)**			
**Noradrenaline**			
(E-) control	5.92 ± 0.10	14.37 ± 3.13	3/5
(E-) 6-CYD (10 pM)	5.80 ± 0.12	15.23 ± 2.57	3/5
(E-) control	5.94 ± 0.10	19.94 ± 2.11	3/6
(E-) 6-CYD (100 pM)	5.86 ± 0.11	28.90 ± 3.04*	3/6
**Adrenaline**			
(E-) control	5.94 ± 0.10	17.57 ± 4.08	4/6
(E-) 6-CYD (100 pM)	5.87 ± 0.11	13.40 ± 0.92	4/6
(E-) control	5.91 ± 0.11	14.38 ± 3.10	4/5
(E-) 6-CYD (1 nM)	6.02 ± 0.10	12.45 ± 1.29	4/5
**Dopamine**			
(E-) control	4.40 ± 0.18	13.30 ± 3.94	3/6
(E-) 6-CYD (100 pM)	4.72 ± 0.10	14.51 ± 1.60	3/6
(E-) control	4.40 ± 0.17	13.30 ± 3.94	3/6
6-CYD (1 nM)	4.87 ± 0.10	11.15 ± 0.75	3/6

Data are expressed as mean ± SEM. The number of experiments is indicated by *x*/*y* where *x* represents the number of animals and *y* the number of rings

^*^p < 0.05 compared with the control group

Adrenaline (0.01–300 μM) elicited concentration-dependent contractions in endothelium-intact aortic rings (Fig. 2B, D, and F). Pre-incubation with 6-CYD at 1 pM for 30 min produced no change in the concentration–response curve to adrenaline (Fig. 2B). However, pre-incubation with 6-CYD at 10 pM (Fig. 2F) and 100 pM (Fig. 2F) significantly potentiated adrenaline-induced contractions, as demonstrated by an increase in the maximal contractile response (*E*_max_) compared with control rings, without significantly changing the pEC_50_ values (Table 1).

### Effect of 6-CYD on noradrenaline- and adrenaline-induced contractions in aortic rings denuded of endothelium

**Fig. 3:**
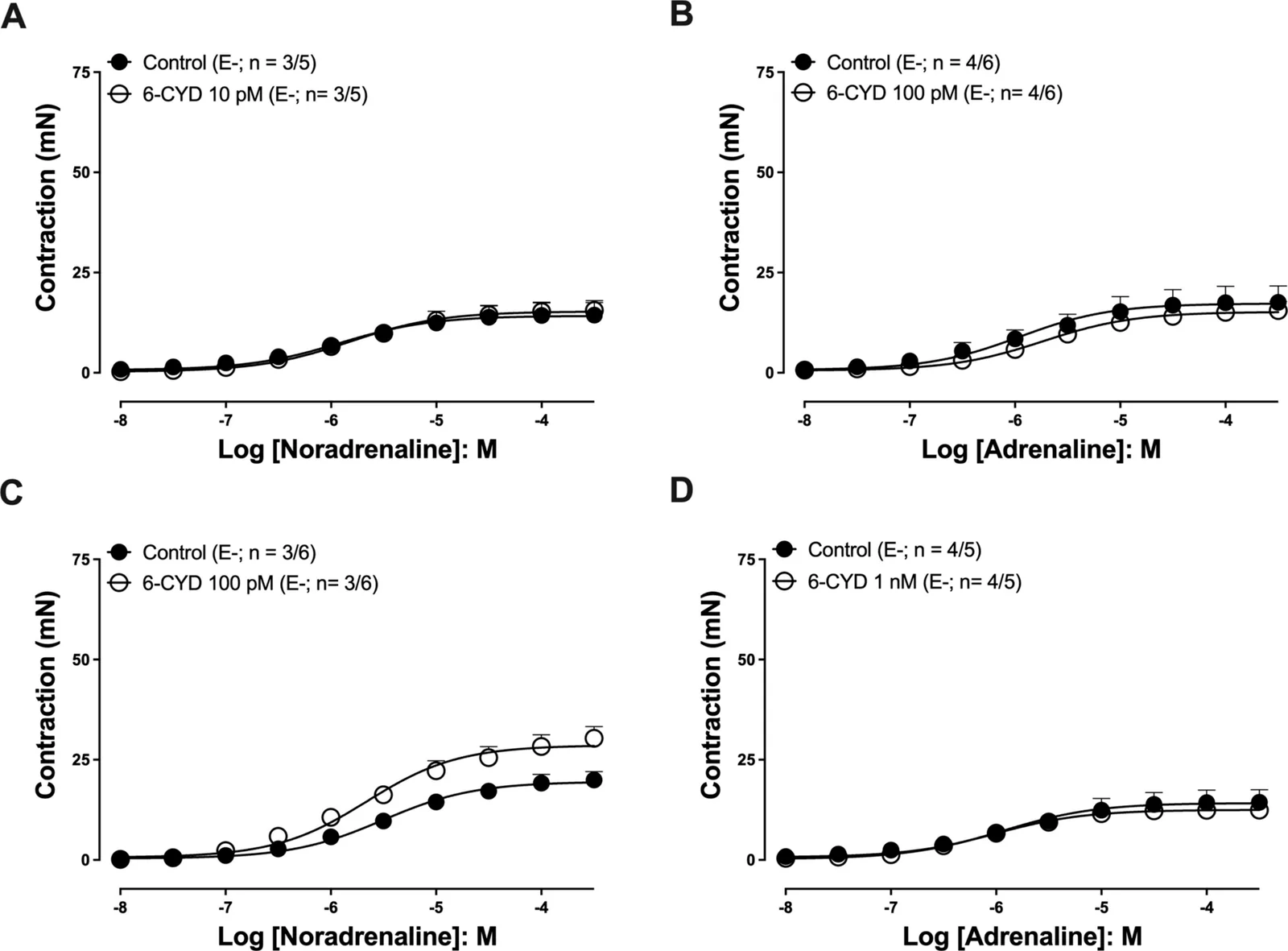
Effects of 6-cyanodopamine (6-CYD) on noradrenaline- and adrenaline-induced contractions in endothelium (E^−^)-denuded rabbit aortic rings. Concentration–response curves to noradrenaline (**A**, **C**) and to adrenaline (**B**, **D**). Rings were incubated for 30 min with 6-CYD at 10 pM (**A**), 100 pM (**B**, **C**), or 1 nM (**D**). Control responses were obtained in the absence of 6-CYD. Data are expressed as mean ± SEM. The number of experiments is indicated by *x*/*y* where *x* represents the number of animals and *y* the number of rings

In endothelium-denuded aortic rings, noradrenaline (0.01–100 μM) also elicited concentration-dependent contractions under control conditions (Fig. 3A and C), despite such contractions being significantly reduced compared with endothelium-intact preparations. Pre-incubation of the rings for 30 min with 6-CYD at 10 pM produced no modification of the noradrenaline concentration–response curve (Fig. 3A). In contrast, pre-incubation with 6-CYD at 100 pM significantly potentiated noradrenaline-induced contractions, as evidenced by an increase in the maximal contractile response (Fig. 3C), without alteration in the pEC_50_ (Table 1). In the same experimental preparation, the contractions induced by adrenaline (0.01–300 μM) were not modified by pre-incubation with 6-CYD at either 100 pM or 1 nM (Fig. 3B and D, respectively). Although the pEC_50_ for noradrenaline and adrenaline was similar in aorta with and without the endothelium, the *E*_max_ was significantly lower in the endothelium-denuded aorta.

### Effect of 6-CYD on dopamine-induced contractions in aortic rings

**Fig. 4:**
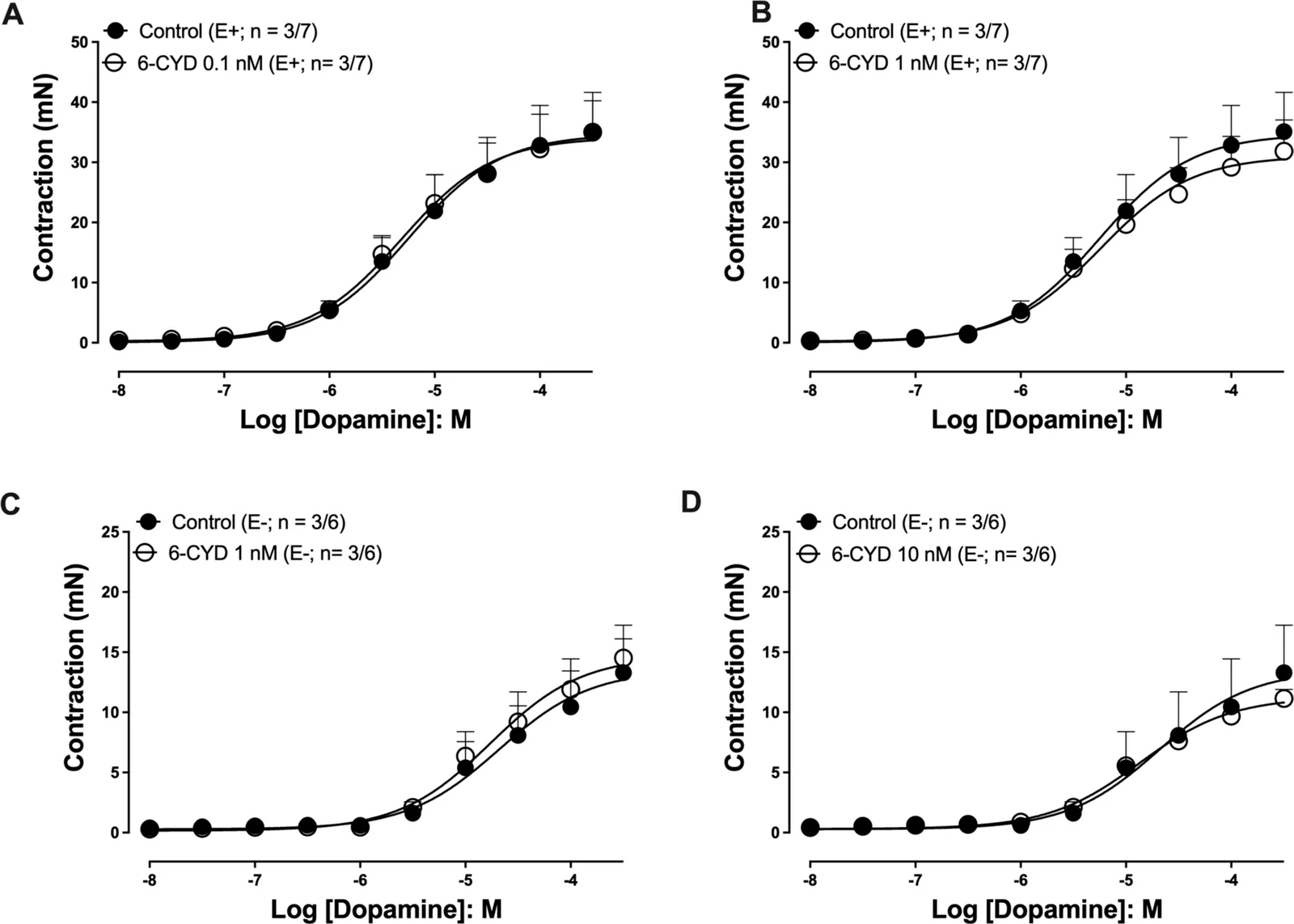
Effects of 6-cyanodopamine (6-CYD) on dopamine-induced contractions in rabbit aortic rings with intact- (**A**, **B**) or denuded-endothelium (**C**, **D**). Concentration–response curves to dopamine are shown (**A**–**D**). Rings were incubated for 30 min with 6-CYD at 100 pM (**A**), 1 nM (**B**, **C**), or 10 nM (**D**). Control responses were obtained in the absence of 6-CYD. Data are expressed as mean ± SEM. The number of experiments is indicated by *x*/*y* where *x* represents the number of animals and *y* the number of rings

Dopamine (0.01–300 μM) induced contractions in both endothelium-intact (Fig. 4A and B) and endothelium-denuded aortic preparations (Fig. 4C and D). Pre-incubation of the endothelium-intact aortic rings with 6-CYD at 0.1 and 1 nM (Fig. 4A and B, respectively) did not affect the contractions induced by dopamine. Similarly, in endothelium-denuded aortic rings, pre-incubation with 6-CYD at the same concentrations (Fig. 4C and D, respectively) also failed to alter dopamine-induced contractions. The pEC_50_ and *E*_max_ values are reported in Table 1.

### Relaxing effects of 6-CYD on pre-contracted aortic rings

**Fig. 5:**
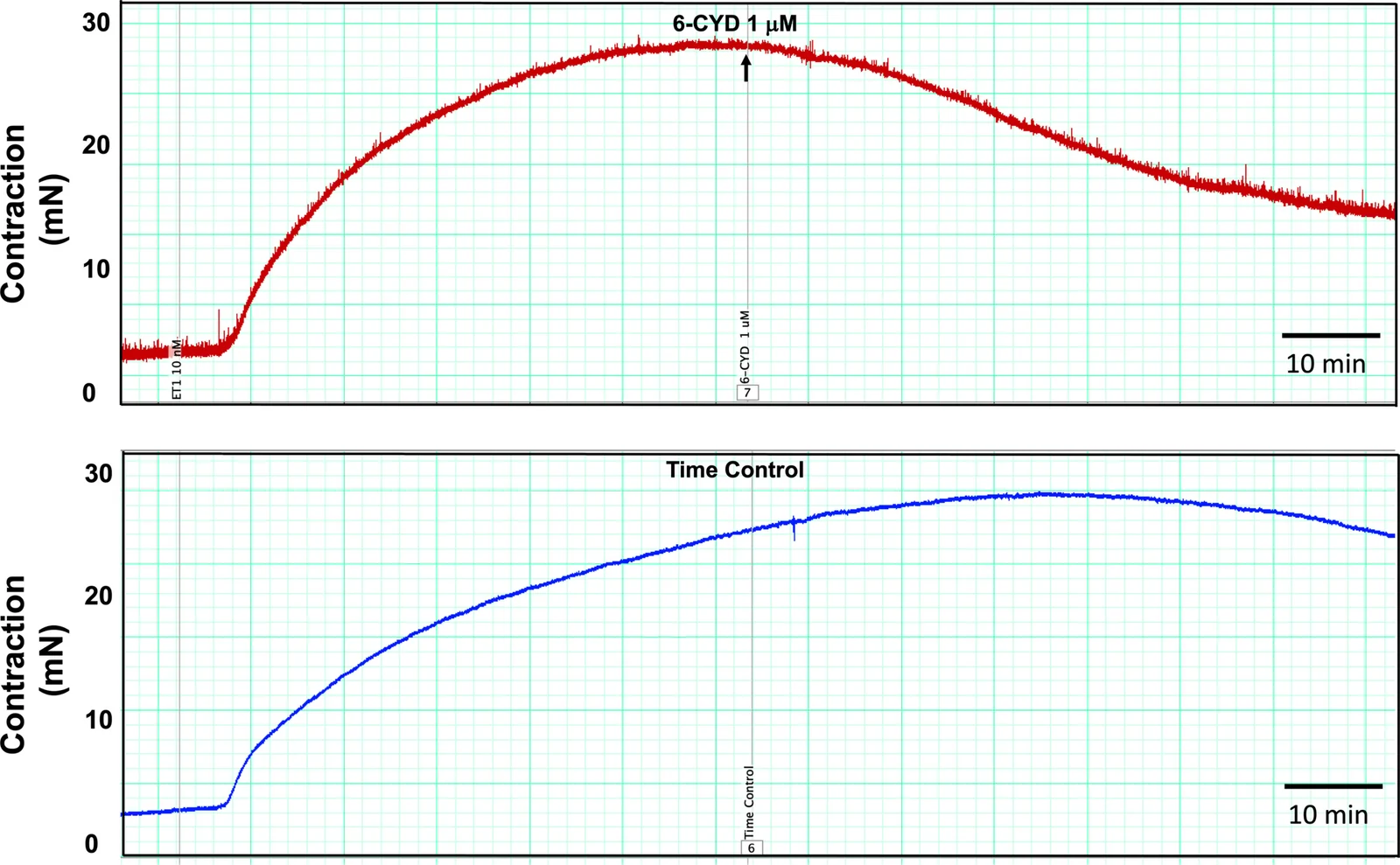
Representative tracings showing the relaxant effect of 6-cyanodopamine (6-CYD) on rabbit isolated aorta rings pre-contracted with endothelin-1 (ET-1). ET-1 (10 nM) induced sustained aortic contraction, after which the addition of 6-CYD (1 µM) produced a marked relaxation response (**A**). Time-control tracing showing the sustained contraction induced by ET-1 (10 nM) in the absence of 6-CYD (**B**)

In endothelium-intact aortic rings, ET-1 (10 nM) induced slow contractions, reaching a maximal response at approximately 90 min (Fig. 5A and B). The addition of 6-CYD at 1 µM produced a clear relaxant effect, reducing by approximately 50% the contractile tone (Fig. 5A). In time-control (Fig. 5B) experiments, no fall in tonus was observed.

### Endothelium dependence and involvement of the NO/cGMP pathway in 6-CYD-induced aortic relaxations

**Fig. 6:**
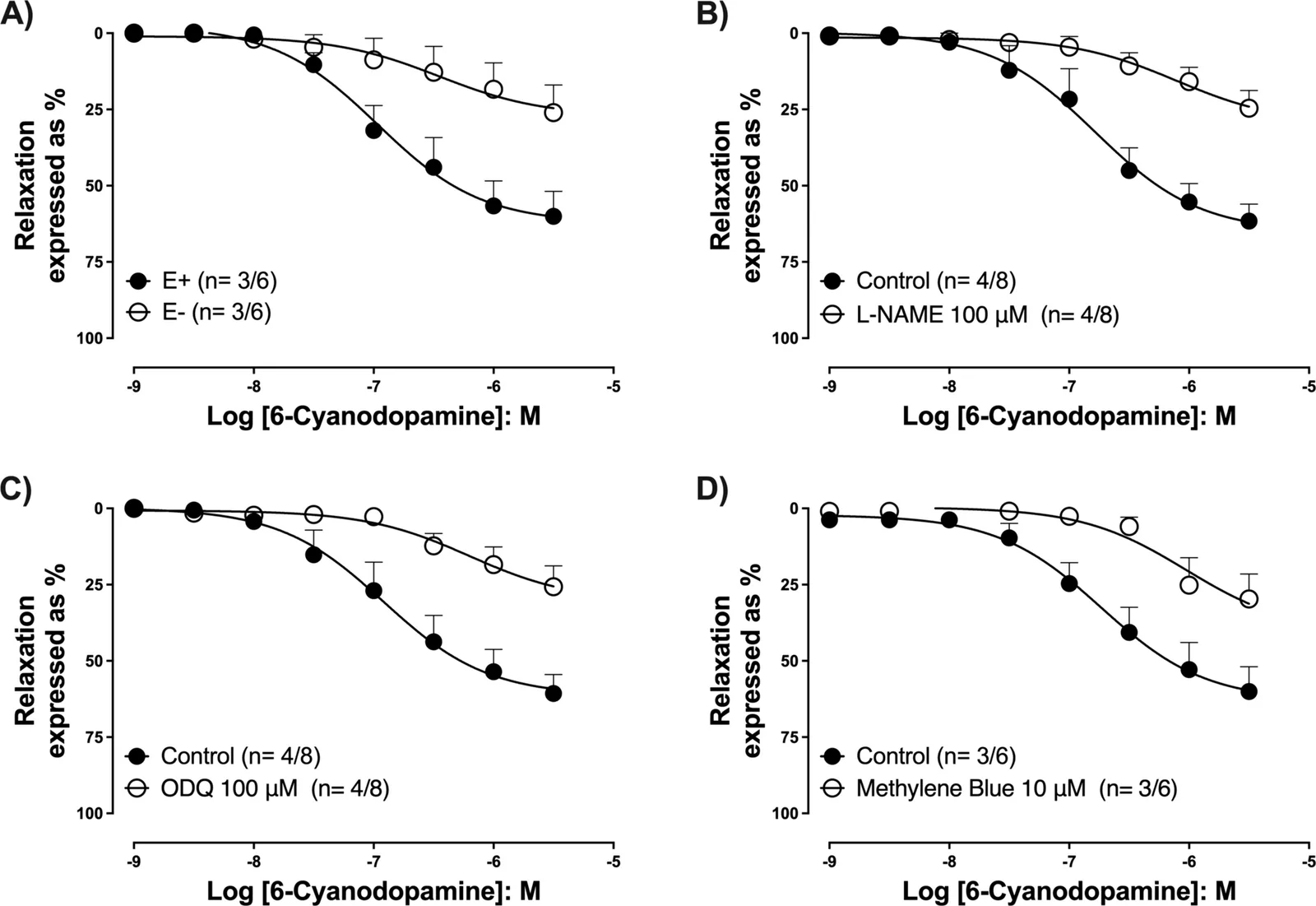
Endothelium dependence and involvement of the NO/cGMP pathway in 6-cyanodopamine-induced relaxation of rabbit isolated aortic rings. Concentration–response curves to 6-cyanodopamine (1 nM–10 µM) were obtained in rabbit aortic rings pre-contracted with endothelin-1 (ET-1, 10 nM). Comparison between endothelium-intact (E⁺) and endothelium-denuded (E⁻) rings is shown (**A**). The effects of pre-incubation for 30 min with L-NAME (100 µM), ODQ (100 µM), and methylene blue (10 µM) in E^+^ preparations are shown (**B**–**D**, respectively). Data are expressed as mean ± SEM. The number of experiments is indicated by *x*/*y* where *x* represents the number of animals and *y* the number of rings

In endothelium-intact aortic rings, 6-CYD (0.001–10 μM) induced concentration-dependent relaxations in aortic rings pre-contracted with ET-1 (10 nM), achieving pEC_50_ and *E*_max_ values of 6.8 ± 0.1 and 62 ± 4%, respectively (Fig. 6A–D). The 6-CYD-induced relaxations were significantly reduced when the endothelium was mechanically removed (Fig. 6A), or when the aortic rings were pre-treated with L-NAME (100 μM; Fig. 6B), ODQ (100 μM; Fig. 6C), or methylene blue (10 μM; Fig. 6D).

## Discussion

This is the first demonstration that isolated vascular tissue presents endothelium-dependent basal release of 6-CYD. In the endothelium-intact aortic rings, the NOX1/4 inhibitor GKT137831 also abolished 6-CYD release whereas L-NAME and TTX failed to affect the 6-CYD release. NADPH oxidase 4 (NOX4) primarily generates hydrogen peroxide, and this reactive oxygen species has been implicated in 6-CYD biosynthesis (Zgiczynski and Stelmaszynska, 1979). However, it is unlikely that this mechanism is responsible for the inhibition of 6-CYD biosynthesis by GKT137831, since pre-incubation of rat isolated ventricles with H_2_O_2_ caused significant reduction in 6-CYD release (Britto-Júnior et al. 2025).

We next evaluated the contractile and relaxant properties of 6-CYD, and the importance of endothelium to modulate both activities. The finding that 6-CYD per se did not contract the aortic rings, even under NO synthesis inhibition, is not surprising. In both human (Britto-Júnior et al. 2026a) and rat (Dal Pozzo et al. 2024) isolated vas deferens, 6-CYD also failed to contract the smooth muscle (in the rat vas deferens, a small contraction was observed only at 1 mM). However, at lower concentrations, 6-CYD potentiated the vasoconstrictions induced by noradrenaline and adrenaline, without affecting that induced by dopamine. Although 6-CYD is known to potentiate the actions of the classical catecholamines dopamine, noradrenaline, and adrenaline in rat isolated heart (Britto-Júnior et al. 2025) and rat and human isolated vas deferens (Dal Pozzo et al. 2024; Britto-Júnior et al. 2026a), the potentiation here reported has some intriguing characteristics. In the vas deferens, 6-CYD is 10 times more potent to increase noradrenaline-induced contractions as compared to dopamine-induced contractions. However, as reported here, even at 10 nM, no potentiation of dopamine-induced contractions was observed, whereas at 1 pM, a substantial increase in the noradrenaline-induced contractions was detected.

In the isolated heart, 6-CYD shares the same properties of 6-ND; i.e., it has positive chronotropic and inotropic effects and potentiates the effects of the classical catecholamines dopamine, noradrenaline, and adrenaline (Britto-Júnior et al. 2025). However, in the rabbit aortic rings, there are some important differences in the actions of 6-CYD as compared to 6-ND. For instance, endothelium-derived 6-ND is the most potent endogenous relaxant of the rabbit isolated aorta, and although the mechanism was endothelium-dependent, it did not involve the NO-sGC-cGMP pathway (Santos et al. 2024). As shown here, vasorelaxation induced by 6-CYD was only observed at higher concentrations, and it was clearly dependent on NO synthesis, since it was strongly reduced by pre-treatment of the aortic rings with L-NAME (Rees et al. 1990), or by pre-incubation with either the nitric oxide-activated soluble guanylyl cyclase inhibitor ODQ (Garthwaite et al. 1995) or with the non-specific soluble guanylyl cyclase inhibitor methylene blue (Gruetter et al. 1981). Sodium cyanide at 10^−5^ M and 10^−3^ M produced relaxation of noradrenaline pre-contracted rabbit aorta (Robinson et al. 1985), but the mechanism was not investigated. Although the 6-CYD concentration to cause relaxation is more than hundred times higher than that to elicit the potentiating effect on catecholamines, extrapolating levels from in vitro studies to assess in vivo relevance may be controversial (Warner et al. 1989). Since 6-CYD is released by the endothelium, the abluminal concentration could be quite substantial as compared to the adluminal concentration.

α_1_-Adrenoceptors are classical adrenoceptors mediating vascular smooth muscle contractions to catecholamines (Docherty 2010). They are divided into α_1A_-, α_1B_-, and α_1D_-adrenoceptors, all of which mediate contractile responses involving Gq/11 and inositol phosphate turnover (Hieble et al. 1995). Prazosin induces a significant rightward displacement of the noradrenaline concentration–response curve in the rabbit isolated aorta (Muramatsu et al. 1990), supporting the concept that noradrenaline causes vasoconstriction via α_1_-adrenoceptor activation. Which α_1_-adrenoceptor is involved is not clear, since significant heterogeneity exists in α_1_-adrenoceptor subtypes in the rabbit aorta (Babich et al. 1987; Auguet et al. 1995). The finding that pEC_50_ of both noradrenaline and adrenaline is basically the same in the rabbit aorta indicates that they are acting on the same α_1_-adrenoceptor. Consistently, in the rat primary motor cortex, the effects of dopamine on gamma power were blocked by prazosin (Özkan et al. 2017). In carbachol pre-contracted rabbit aorta, incubation with the non-selective β-adrenoceptor antagonist propranolol inhibited the relaxations induced by both dopamine and noradrenaline (Kohli 1969).

Although dopamine-induced vasoconstriction of the rat isolated aorta is supposed to be mediated by α_1_-adrenoceptor activation (Lee et al. 2026), it is important to note that all five dopamine receptors have been identified in vascular beds in vitro by radioligand binding, autoradiographic techniques, and immunohistochemical analysis (Amenta et al. 1990). Drugs that target GPCRs can be promiscuous in binding activity between related GPCRs. Indeed, dopamine lacks selectivity, stimulating both dopamine receptor subtypes and α- and β-adrenoceptors, which limits the clinical usefulness of dopamine (Goldberg, 1974; Makabali et al. 1982). The non-selectivity is also observed with antagonists. For instance, the dopamine D_2_-like antagonists do interact with adrenergic receptors, and the differences in potency (ki) for haloperidol (1.4 and 4.7 nM, for D_2_ and α_1_-adrenoceptor, respectively) and risperidone (2.2 and 1.4 nM, for D_2_ and α_1_-adrenoceptor, respectively) are discrete (Schmidt et al. 2001; Campiani et al. 2002). Similarly, α_1_-adrenergic antagonists do interact with dopaminergic receptors at low concentrations; tamsulosin binds to D_2_ and D_3_ receptors at 16 nM and 8 nM, respectively (McLeod-Flynn 2009), and prazosin binds to D_3_ receptor (Zhang et al. 2012). The finding here, which reported that 6-CYD potentiated the contractions induced by both noradrenaline and adrenaline, but not that induced by dopamine, indicates that dopamine is not acting on α_1_-adrenoceptors. Another piece of evidence that the contraction induced by dopamine is the result of dopaminergic receptor activation is the finding that 6-ND selectively blocks the contraction of marmoset aorta and pulmonary artery rings induced by dopamine, without affecting those induced by noradrenaline and adrenaline (Britto-Júnior et al. 2023). It is interesting that pre-treatment of human aortic smooth muscle cells with tetrodotoxin blocks the increase in intracellular calcium induced by 6-ND and dopamine, but not that caused by noradrenaline and adrenaline, indicating that the former catecholamines may interact with voltage-gated sodium channels (Britto-Júnior et al. 2026b). Whether the potentiation induced by 6-CYD also involves activation of sodium channels is under current investigation.

The mechanism by which 6-CYD potentiates the contractions of both noradrenaline and adrenaline in the rabbit isolated aorta clearly depends on endothelium integrity. Although the pEC_50_ for noradrenaline and adrenaline was similar in the aorta with and without the endothelium, the *E*_max_ was significantly lower in the endothelium-denuded aorta, indicating that these catecholamines not only act at the vascular smooth muscle to induce contractions but also stimulate endothelium to release contractile factor(s). Similar results were observed for angiotensin II and noradrenaline in the rabbit aortic rings, where the pD_2_ for angiotensin II and noradrenaline was not affected by removal of the endothelium, but the maximum effect was greater in the endothelium-intact aortae (Jerez et al. 2004). It is interesting that in pig and dog isolated coronary arteries, noradrenaline was significantly a more powerful vasoconstrictor in the absence of endothelium (Cocks & Angus 1983). It is possible that this ability of the endothelium to potentiate noradrenaline-induced contractions may depend on the vascular bed.

Endothelial cells express β- (Steinberg et al. 1984; Summers et al. 1987) and α_1_- (McGrath 2015) and α_2_- (Guimarães & Moura 2001) adrenoceptors. Although their presence has been associated with vasodilator responses (Miller and Vanhoutte 1985; Angus et al. 1986), this piece of evidence is based essentially on endothelium β- and α_2_-adrenoceptors. The finding that 6-CYD-induced potentiation on aortic contractions does not occur with dopamine suggests that this potentiation is not due to release of an endothelium-derived contractile factor (Dhein et al. 1989). It is interesting that adenylyl cyclase–associated proteins have been identified as potential receptors for 6-ND in human cardiomyocytes (Cipollone et al. 2025). 6-Nitrodopamine increases intracellular calcium levels in human aortic endothelial cells (Britto-Júnior et al. 2025). Whether 6-CYD modulates intracellular calcium levels in endothelial cells is under current investigation.

## Supplementary Information

Below is the link to the electronic supplementary material.

(PNG 39.9 KB)

ESM 1 Figure S1. Lack of effect of 6-cyanodopamine (6-CYD) on rabbit aortic rings with intact endothelium (E+) in the presence of L-NAME (100 μM). Data are expressed as mean ± SEM. The number of experiments is indicated by x/y where x represents the number of animals and y the number of rings. (TIFF 244 KB)

## Data Availability

All source data for this work (or generated in this study) are available upon reasonable request.
